# HIV, syphilis, and gonorrhea co-infection and associated factors among adolescents and young adults aged 15–24 years in Ningbo, China, 2005–2024

**DOI:** 10.3389/fpubh.2025.1702301

**Published:** 2026-01-12

**Authors:** Haibo Jiang, Xing Zhang, Lixia Ye, Shiwen Tan, Kun Chu, Zehao Ye, Zhixin Zhu, Chengliang Chai, Yi Chen

**Affiliations:** 1 Ningbo Municipal Center for Disease Control and Prevention, Ningbo, Zhejiang, China; 2 Zhejiang Association of STD/AIDS Prevention and Control, Hangzhou, Zhejiang, China; 3 Quzhou Center for Disease Control and Prevention, Quzhou, Zhejiang, China; 4 Zhejiang Provincial Center for Disease Control and Prevention, Hangzhou, Zhejiang, China

**Keywords:** HIV/AIDS, syphilis, gonorrhea, adolescents, co-infection, sexually transmitted infections, trend, associated factors

## Abstract

**Background:**

Sexually transmitted infections (STIs), including HIV, syphilis, and gonorrhea, have exhibited a rising trend among adolescents and young adults aged 15–24 years in China, presenting a major public health concern. This age group faces heightened risks due to behaviors such as unprotected sex, having multiple partners, and engaging in online dating, as well as limited access to healthcare services. Monitoring long-term trends and factors associated with STI co-infections is essential for developing effective prevention and intervention strategies.

**Methods:**

Historical data on newly reported syphilis and gonorrhea cases in Ningbo from 1 January 2005 to 31 December 2024 were retrieved from the China Disease Prevention and Control Information System. Cases with final review dates between 2005 and 2024 that were reported in Ningbo were selected and matched with concurrent case data from the Comprehensive HIV/AIDS Prevention and Control Information System to investigate factors influencing co-infections with two or more STIs (specifically syphilis, gonorrhea, or HIV) among individuals aged 15–24 years. Chi-squared tests for trend were employed to assess temporal patterns in HIV, syphilis, and gonorrhea epidemics, while multivariate logistic regression models were used to evaluate factors associated with STI co-infections.

**Results:**

From 2005 to 2024, the total reported cases of HIV, syphilis, and gonorrhea among adolescents and young adults aged 15–24 years in Ningbo were 1,576 (6.0%), 14,623 (55.4%), and 10,204 (38.6%), respectively. The reported epidemics of HIV, syphilis, and gonorrhea in this demographic exhibited a significant upward trajectory over the period (linear regression analysis, *p* < 0.05). Over the 20-year span, the reported HIV incidence rate demonstrated an overall increasing trend (trend *χ*^2^ = 11.735, *p* < 0.001). The reported prevalence of HIV, syphilis, and gonorrhea co-infections in this population was 4.9% (1,304/26,403). Significant predictors of STI co-infections included the reporting year (2015–2024 vs. 2005–2014, aOR = 11.637, 95% CI: 9.740–13.905), sex, occupation, household registration, and sample source (*p* < 0.05).

**Conclusion:**

Targeted interventions should prioritize male individuals, those in high-risk situations (such as those engaged in housework, unemployed, incarcerated, working in commercial services, students, or medical personnel), and those with non-local (out-of-province) household registration through enhanced STI health education, behavioral interventions, and expanded provider-initiated HIV testing and counseling (PITC) to curb the rising trends in HIV and STI epidemics.

## 1 Introduction

In recent years, the incidence of sexually transmitted infections (STIs), such as HIV/AIDS, syphilis, and gonorrhea, has exhibited a marked upward trend among adolescents and young adults aged 15–24 years. This trend is particularly evident in China and requires heightened attention from public health stakeholders (1). Data obtained from the National Notifiable Disease Reporting System indicate that newly diagnosed HIV cases in China rose from 9,373 in 2010 to 15,790 in 2019, reflecting an average annual growth rate of 6.0% (2). From 2010 to 2020, a total of 128,646 HIV/AIDS cases were reported nationwide among out-of-school youth aged 15–24 years, with the crude reporting rate increasing from 5.25 per 100,000 in 2010 to 13.75 per 100,000 in 2020 (3). This rise not only reflects improvements in testing coverage (4) but is also strongly linked to increased high-risk behaviors, including condomless sex and multiple sexual partners (5).

Moreover, the prevalence of STI co-infections in this demographic is on the rise, particularly the co-occurrence of HIV with syphilis or gonorrhea. This rise may intensify transmission dynamics and complicate treatment efforts (6). Evidence suggests that HIV/AIDS and syphilis co-infection rates among adolescents and young adults have surged over the past decade, especially in high-risk subgroups such as men who have sex with men (7). However, investigations into social networking patterns (e.g., online dating) (8) and infection risk factors among out-of-school youth remain limited (9), with current targeted interventions, testing, and educational initiatives providing insufficient coverage, thereby hindering the formulation of robust prevention strategies (10). For example, epidemiological surveys focused on out-of-school adolescents are scarce (11), and there is a dearth of targeted research on migrant populations and unemployed youth (12), highlighting the urgent need for studies on tailored prevention strategies to inform more effective intervention frameworks (13). This study used historical surveillance data from Ningbo to analyze the epidemiological profiles and risk factors for HIV, syphilis, and gonorrhea among adolescents and young adults aged 15–24 years, with the aim of providing scientific evidence for prevention and control measures (14).

Ningbo, as a representative coastal city in China, warrants focused scrutiny of STI infections in its adolescent and young adult population. The primary objective of this study was to delineate the epidemiological trends of HIV, syphilis, and gonorrhea among individuals aged 15–24 years in Ningbo from 2005 to 2024. In addition, the study aims to elucidate the associated factors contributing to co-infections. By conducting longitudinal tracking and analyzing infection data in this cohort, the study sought to offer foundational references for developing evidence-based intervention measures and prevention strategies.

## 2 Methods

### 2.1 Study participants

The inclusion criteria for this study are as follows: (1) newly diagnosed cases of HIV/AIDS, syphilis, or gonorrhea in Ningbo from 1 January 2005 to 31 December 2024. (2) In accordance with the requirements of the Law of the People’s Republic of China on the Prevention and Treatment of Infectious Diseases, informed consent was not required, and all confirmed STI cases were mandated to participate in the investigation.

### 2.2 Data source

A cross-sectional epidemiological survey was conducted. Trained personnel from the Center for Disease Control and Prevention carried out one-on-one, face-to-face interviews with participants after obtaining informed consent, utilizing the People’s Republic of China Notifiable Disease Reporting Card and a supplementary HIV/STI questionnaire. The questionnaire collected information on general demographics, high-risk behavior history, HIV testing history, local residency history, transmission routes, and receipt of relevant intervention services.

### 2.3 Relevant definitions

The yearly reported incidence rate of STIs was defined as the number of new HIV, syphilis, or gonorrhea cases reported in Ningbo (after excluding duplicates) divided by the annual resident population aged 15–24 years. The numerator was sourced from the China Information System for Disease Control and Prevention (CISDCP), based on reports from Ningbo. The denominator population data were obtained from the age statistics module of the Chinese infectious disease monitoring system.

STI co-infection was defined as the concurrent infection with two or more pathogens among newly reported STI cases, including HIV, syphilis, or gonorrhea, where the “confirmation detection positive date” variables for the respective pathogens (AIDS, syphilis, and gonorrhea) were the same, as confirmed by positive laboratory results (e.g., serological tests for syphilis/HIV and nucleic acid tests for gonorrhea) at diagnosis.

Sources of blood samples for newly diagnosed HIV/AIDS cases (hereafter “sample sources”) were classified as either provider-initiated HIV testing and counseling (PITC) or voluntary HIV counseling and testing (VCT).

### 2.4 Statistical analysis

Excel 2019 was utilized to stratify case data by diagnosis year, general demographic characteristics, sample sources, and transmission routes to evaluate STI co-infection trends. The data were then imported into the R 4.5.1 software. Temporal variations in the reported HIV, syphilis, and gonorrhea epidemics were assessed using linear regression models, while changes in STI co-infection prevalence and reported incidence rates were examined using chi-squared tests for trend. Univariate analyses and multivariate logistic regression were conducted to assess STI co-infection status, using grouping variables such as general demographic information, sexual contact history, transmission routes, and sample sources. Odds ratios (ORs) and 95% confidence intervals (CIs) were calculated to quantify the associations between independent variables and the outcome. All tests were two-tailed, with statistical significance set at a *p*-value of <0.05.

### 2.5 Data quality control and deduplication

To address potential reporting bias in long-term retrospective data, all cases were extracted from the China Information System for Disease Control and Prevention (CISDCP), which uses national ID-based automatic deduplication. Syphilis and gonorrhea cases were included only if the final review status was “confirmed” and the cases were reported in Ningbo. HIV cases from the Comprehensive HIV/AIDS Prevention and Control Information System (CRIMS) were merged using the same unique national ID and final review date. Data cleaning was performed independently by two senior epidemiologists (HJ and XZ) using R 4.5.1. Discrepancies were resolved by consensus with a third investigator (LY). Cases with missing national IDs or conflicting review dates were excluded (*n* = 41, 0.16%). Reporting completeness for syphilis/gonorrhea improved from ~70% in 2005–2010 to >95% after 2015, following national training programs (15, 16).

## 3 Results

### 3.1 Demographic and behavioral characteristics

From 2005 to 2024, the reported cases of HIV, syphilis, and gonorrhea among adolescents and young adults aged 15–24 years in Ningbo totaled 1,576 (6.0%), 14,623 (55.4%), and 10,204 (38.6%), respectively. The mean age of the reported cases was 21.2 ± 2.3 years, with 12,806 male individuals (48.5%), 25,559 of Han ethnicity (96.8%), 16,185 having a junior high school education or below (61.3%), 22,672 unmarried (85.9%), 24,987 currently residing in Ningbo (94.6%), 22,416 with household registration in Ningbo (84.9%), and occupations primarily consisting of housework and unemployment (7,951, 30.1%), farmers (5,055, 19.1%), and workers (2,694, 10.2%), including 885 students (3.4%).

The reported cases included 2,024 individuals with a history of heterosexual commercial sex (7.7%), 949 with a history of male–male sexual contact (3.6%), and 271 with a history of heterosexual non-commercial sex (1.0%). The number of non-marital heterosexual partners was ≥2 for 733 individuals (2.8%), the number of male–male sexual partners was ≥2 for 778 individuals (2.9%), and the number of shared injection drug use partners was ≥1 for 26 (0.1%). The most likely transmission route was heterosexual for 2,204 cases (8.3%), with the sample sources being provider-initiated testing and counseling (PITC) for 25,903 individuals (98.1%) (see Table 1).

**Table 1:** Univariate analysis of general demographic characteristics and STI co-infection among adolescents aged 15–24 years in Ningbo.

Variables	Number of cases (*n*)	STI co-infection		Univariate analysis	
		Number of cases	Proportion (%)	*χ*^2^ value	*p*-value
Year				1487.943	<0.001
2005	1,416	1	0.1		
2006	1,384	4	0.3		
2007	1,659	10	0.6		
2008	1,916	4	0.2		
2009	1,777	13	0.7		
2010	1,807	34	1.9		
2011	1,735	19	1.1		
2012	1,285	13	1.0		
2013	1,162	18	1.5		
2014	1,060	29	2.7		
2015	1,075	42	3.9		
2016	1,073	140	13.0		
2017	1,229	172	14.0		
2018	1,249	139	11.1		
2019	1,304	163	12.5		
2020	1,098	145	13.2		
2021	1,208	145	12.0		
2022	1,131	97	8.6		
2023	970	60	6.2		
2024	865	56	6.5		
Time period (year)				1211.847	<0.001
2005–2014	15,201	145	1.0		
2015–2024	11,202	1,159	10.3		
Sex				4.311	0.038
Male individuals	12,806	669	5.2		
Female individuals	13,597	635	4.7		
Age				33.102	<0.001
15 ~ 19	6,291	397	6.3		
20 ~ 24	20,112	907	4.5		
Current residence (in the past 6 months)				48.167	<0.001
Ningbo	24,987	1,183	4.7		
Other cities in Zhejiang province	300	17	5.7		
Other provinces of China	1,116	104	9.3		
Occupation				132.615	<0.001
Catering and food industry/public venue attendants/workers/farmers/herdsmen/migrant workers/seafarers and long-distance drivers/fishermen (boatmen)/childcare workers and nannies/individual merchants/cadres and staff/teachers/retired personnel/scattered children	15,039	542	3.6		
Housework and unemployed/detained personnel/commercial services/students/medical personnel	11,364	762	6.7		
Marital status				58.574	<0.001
Unmarried	22,672	1,028	4.5		
Divorced or widowed	65	2	3.1		
Married with spouse	3,666	274	7.5		
Ethnicity				0.828	0.406
Han	25,559	1,150	4.5		
Other ethnicities	844	44	5.2		
Education level				0.185	0.911
Junior high school and below	16,185	858	5.3		
High school or technical secondary school	5,077	249	4.9		
Junior college and above	5,141	226	4.4		
Household registration				188.687	<0.001
Ningbo	22,416	935	4.2		
Other cities in Zhejiang Province	408	41	10.0		
Other provinces of China	3,552	327	9.2		
Hong Kong/Macau/Taiwan/Foreign	27	1	3.7		
Sexual contact history				169.200	<0.001
Male–male sexual behavior history	949	124	13.1		
Spouse/fixed partner positive	195	21	10.8		
Heterosexual commercial behavior history	2,024	118	5.8		
Heterosexual non-commercial behavior history	271	22	8.1		
Other/unknown	22,964	1,019	4.4		
Number of non-marital heterosexual partners				30.221	<0.001
≤1	25,670	1,236	4.8		
>1	733	68	9.3		
Number of male–male sexual partners				152.705	<0.001
≤1	25,625	1,192	4.7		
>1	778	112	14.4		
Number of injection drug use partners				-	0.638
≤0	26,377	1,304	4.9		
>0	26	0	0		
Most likely transmission route				147.714	<0.001
Male–male sexual transmission	945	123	13.0		
Heterosexual transmission	2,204	138	6.3		
Other/unknown	23,254	1,043	4.5		
Blood sample source				29.876	<0.001
PITC	25,902	1,253	4.8		
VCT	501	51	10.2		

### 3.2 Trends in the reported cases and incidence rates of HIV, syphilis, and gonorrhea

**Figure 1:**
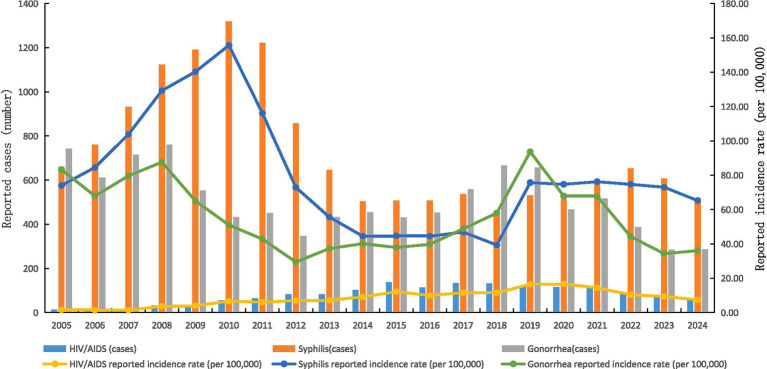
Trends in the reported cases and incidence rates of HIV, syphilis, and gonorrhea among adolescents aged 15–24 years in Ningbo, 2005–2024.

From 2005 to 2024, the co-infection rate of HIV, syphilis, and gonorrhea among adolescents and young adults aged 15–24 years in Ningbo was 4.9% (1,304/26,403). The number of reported HIV cases in this population increased from 14 in 2005 (reported incidence rate: 1.57 per 100,000) to 59 in 2024 (7.42 per 100,000), exhibiting a linear upward trend according to linear regression analysis (*F* = 16.071, *p* = 0.001). In addition, the reported HIV incidence rate showed an increasing trend (trend *χ*^2^ = 11.735, *p =* 0.001). The number of reported syphilis and gonorrhea cases decreased from 660 (74.00/100,000) and 742 (83.20/100,000) in 2005 to 519 (65.23/100,000) and 287 (36.07/100,000) in 2024, respectively. The reported syphilis incidence showed a downward trend (trend *χ*^2^ = 4.437, *p* = 0.035), while the gonorrhea trend was not significant (trend *χ*^2^ = 2.590, *p* = 0.108). This does not reflect a true decline in transmission but likely results from improved deduplication and reporting standardization after 2015 (see Discussion) (see Figure 1).

### 3.3 Co-infection status of HIV, syphilis, and gonorrhea

**Figure 2:**
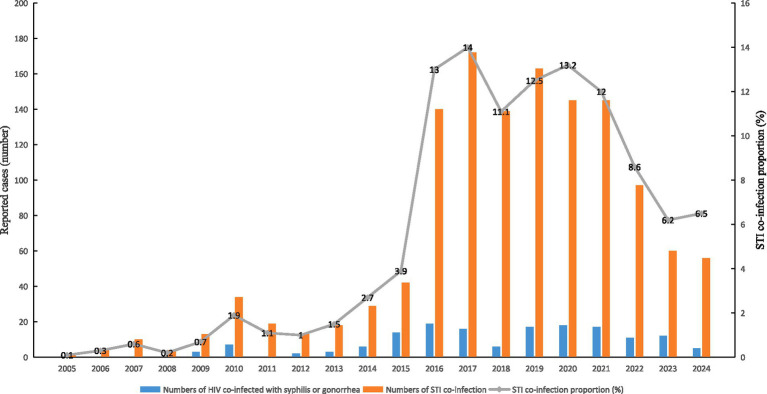
Trends in the co-infection of HIV, syphilis, and gonorrhea among adolescents aged 15–24 years in Ningbo, 2005–2024.

The number of reported HIV cases co-infected with other STIs (one or more) among adolescents and young adults aged 15–24 years increased from 0 in 2005 to 5 in 2024 (*F* = 17.688, *p* = 0.001). The number of co-infections (involving HIV, syphilis, and gonorrhea in two or more combinations) rose from 1 (0.1%) in 2005 to 56 (6.5%) in 2024 (*F* = 17.270, *p* = 0.001), with the co-infection proportion exhibiting an upward trend (trend *χ*^2^ = 10.854, *p* = 0.003) (see Figure 2).

### 3.4 Multivariate analysis of STI co-infection

Using STI co-infection as the dependent variable and demographic characteristics, reporting year, and sample source as independent variables, multivariate logistic regression analysis was performed. The results indicated that reporting year grouping, sex, occupation, household registration, and sample source were statistically significant predictors of STI co-infection (*p* < 0.05). The detailed results are presented in Table 2.

**Table 2:** Multivariate logistic regression analysis of HIV, syphilis, and gonorrhea co-infection among adolescents aged 15–24 years in Ningbo.

Variables	*aOR* value (95%CI)	*p*-value
Time period (year)		
2005–2014	1.000	
2015–2024	11.637 (9.740 ~ 13.905)	<0.001
Sex		
Male individuals	1.000	
Female individuals	1.165 (1.037 ~ 2.310)	0.010
Occupation		
Catering and food industry/public venue attendants/workers/farmers/herdsmen/migrant workers/seafarers and long-distance drivers/fishermen (boatmen)/childcare workers and nannies/individual merchants/cadres and staff/teachers/retired personnel/scattered children	1.000	
Housework and unemployed/detained personnel/commercial services/students/medical personnel	1.310 (1.165 ~ 2.474)	<0.001
Household registration		<0.001
Ningbo	1.000	
Other cities in Zhejiang province	1.398(0.995 ~ 1.965)	0.054
Other provinces of China	1.383(1.205 ~ 1.587)	<0.001
Hong Kong/Macau/Taiwan/Foreign	0.530(0.071 ~ 3.983)	0.537
Blood sample source		
PITC	1.000	
VCT	1.375 (1.015 ~ 3.863)	<0.001

aOR value, adjusted OR value after multiple regression analysis.

## 4 Discussion

Data from the Global Burden of Disease database indicate that the burden of STIs among adolescents and young adults in the Western Pacific region, including China, is increasing. Individuals aged 15–24 years represent a high-risk group for HIV, with syphilis and gonorrhea acting as risk factors for HIV acquisition (17). Based on data from the National Notifiable Disease Reporting System, our findings indicate an increasing incidence of reported HIV, syphilis, and gonorrhea cases among adolescents and young adults aged 15–24 years in Ningbo, consistent with trends observed in other municipalities (18). This escalation may primarily be attributed to a significant increase in HIV testing among this population, including higher testing among STI clinic attendees and expanded coverage across the general population (19). However, due to the lack of comprehensive multi-year data on HIV testing volumes in the 15–24 age group, further research is recommended to explore the associations between HIV testing strategies and epidemic growth in this demographic (20) to better elucidate the characteristics and underlying causes of epidemic changes.

This study found that the reported HIV incidence rate among adolescents and young adults aged 15–24 years in Ningbo increased from 1.57 per 100,000 in 2005 to 7.42 per 100,000 in 2024 (trend *χ*^2^ = 11.735, *p* = 0.001). This rise in HIV incidence may be linked to the growing prevalence of online dating (21), although this study did not specifically investigate dating patterns; future studies should incorporate such assessments to better understand transmission mechanisms. The reported incidence of syphilis showed a downward trend from 2005 to 2024, while gonorrhea followed a similar but non-significant pattern. This contrasts with national upward trends and does not indicate reduced transmission; rather, it likely reflects improved reporting quality. For example, duplicate reporting of syphilis decreased from ~25% in 2005–2010 to <5% after 2015 following national training programs and electronic card standardization (15, 16). In addition, a shift in testing venues, such as increasing gonorrhea diagnoses in private clinics (not fully reported to the CISDCP), may contribute to the apparent decline (22), alongside under-detection of asymptomatic gonorrhea, since routine dual testing is not universally implemented as it is for HIV. These artifacts are common in long-term surveillance and have been reported in Shenzhen and Hangzhou (18, 22), suggesting that the true incidence may be stable or rising. This underscores the need for sentinel surveillance with testing denominators.

The results of this study demonstrate that the co-infection proportion of STIs such as HIV, syphilis, and gonorrhea among adolescents and young adults aged 15–24 years in Ningbo from 2005 to 2024 was 4.9%, aligning with findings from related studies in cities such as Beijing (18, 22). This co-infection prevalence is consistent with levels reported in high-income countries (2–5%) and lower than that observed in low-income countries (5–15%) (17). Although the overall STI co-infection rate in Ningbo is relatively low, both HIV co-infection with other STIs and the multi-STI co-infection rate have shown upward trends, suggesting persistent STI transmission risks in this adolescent and young adult population, with the possibility of undetected latent infections (23). Targeted educational interventions should be implemented to enable the early identification of STI risks among adolescents and young adults.

Through the analysis of reported data, we identified multiple factors closely associated with STI co-infections in this adolescent and young adult population. Demographic characteristics such as reporting year, sex, occupation, and household registration exerted significant effects on STI co-infections. Notably, adolescents and young adults from other provinces, as well as those in high-risk occupations, such as housework and unemployment/detention/commercial services, exhibited higher co-infection proportions. This may be linked to elevated structural risks faced by these groups, including limited access to healthcare and lack of social support (24), consistent with literature on the impact of migration on epidemics (25). These findings imply that interventions should be multi-level, integrating local data to formulate precision-based strategies (26). In addition, this study highlights the importance of provider-initiated testing and counseling (PITC) by healthcare personnel for early STI detection. Promoting joint testing for HIV, syphilis, and gonorrhea in high-risk adolescents and young adults enables timely identification and intervention of infected individuals (4). Strengthening multi-pathogen co-testing and co-prevention strategies is recommended to effectively control STI transmission (17), thereby reducing transmission chains and improving prognosis (2). This evidence supports policy-level promotion, such as multi-disease screening programs at school and community levels (20).

This study has certain limitations, particularly the lack of in-depth epidemiological investigations into dating patterns for syphilis and gonorrhea, which may introduce bias in the assessment of risk factors. Downward trends in syphilis/gonorrhea likely reflect improved deduplication and under-reporting from private sectors rather than reduced incidence. Syphilis confirmation shifted from RPR-only (pre-2010) to TPPA/TPHA, potentially reducing false positives. The absence of testing volume data for syphilis/gonorrhea (unlike HIV) limits the interpretation of the reported incidence. It is recommended to conduct targeted epidemiological surveys among adolescents and young adults aged 15–24 years, including in-depth interviews, to better understand true transmission risk relationships (18). Future research could employ longitudinal cohort designs, incorporate big data analytics to track long-term trends, and expand variable scopes to include rural and remote areas (2). These approaches will provide a robust foundation for developing more effective intervention strategies (27).

## 5 Conclusion

This study examined the epidemiological trends and associated factors of HIV, syphilis, and gonorrhea co-infections among adolescents and young adults aged 15–24 years in Ningbo from 2005 to 2024. The reported HIV incidence showed an upward trend, while syphilis and gonorrhea incidences declined, with an overall co-infection prevalence of 4.9% and increasing multi-STI combinations. Key predictors included reporting year, sex, occupation, household registration, and sample source, highlighting vulnerabilities in high-risk subgroups such as male individuals, non-local residents, and individuals in occupations such as housework or unemployment. These findings underscore the need for targeted interventions, including enhanced STI education, behavioral strategies, and expanded provider-initiated testing and counseling, to mitigate the escalating STI burden in this population.

## Data Availability

The raw data supporting the conclusions of this article will be made available by the authors, without undue reservation.
