# Correction: HIV, syphilis, and gonorrhea co-infection and associated factors among adolescents and young adults aged 15–24 years in Ningbo, China, 2005–2024

**DOI:** 10.3389/fpubh.2026.1788439

**Published:** 2026-02-05

**Authors:** Haibo Jiang, Xing Zhang, Lixia Ye, Shiwen Tan, Kun Chu, Zehao Ye, Zhixin Zhu, Chengliang Chai, Yi Chen

**Affiliations:** 1 Ningbo Municipal Center for Disease Control and Prevention, Ningbo, Zhejiang, China; 2 Zhejiang Association of STD/AIDS Prevention and Control, Hangzhou, Zhejiang, China; 3 Quzhou Center for Disease Control and Prevention, Quzhou, Zhejiang, China; 4 Zhejiang Provincial Center for Disease Control and Prevention, Hangzhou, Zhejiang, China

**Keywords:** HIV/AIDS, syphilis, gonorrhea, adolescents, co-infection, sexually transmitted infections, trend, associated factors

In the published article, there was a mistake in the author affiliations. The affiliation for the second author (Xing Zhang) was erroneously given as “Zhejiang Association of STD/AIDS Prevention and Control, Hangzhou, Zhejiang, China”. The correct affiliation is “Quzhou Center for Disease Control and Prevention, Quzhou, Zhejiang, China”.

The correct and complete author affiliation list should read:

**Haibo Jiang** ^**1,2†**^ **, Xing Zhang** ^**3†**^ **, Lixia Ye** ^**1,2**^ **, Shiwen Tan** ^**1**^ **, Kun Chu** ^**1**^ **, Zehao Ye** ^**1**^ **, Zhixin Zhu** ^**1**^ **, Chengliang Chai** ^2,4*^ **, Yi Chen** ^1*^

^1^Ningbo Municipal Center for Disease Control and Prevention, Ningbo, Zhejiang, China, ^2^Zhejiang Association of STD/AIDS Prevention and Control, Hangzhou, Zhejiang, China, ^3^Quzhou Center for Disease Control and Prevention, Quzhou, Zhejiang, China, ^4^Zhejiang Provincial Center for Disease Control and Prevention, Hangzhou, Zhejiang, China

The funders Zhejiang Provincial Disease Prevention and Control Science and Technology Program, (No. 2026JKZ057) and Ningbo Top Medical and Health Research Program, (No. 2023020713) were erroneously omitted. All authors of the original paper agree to the requests for changes.

The correct Funding statement should read:

The author(s) declared that financial support was received for this work and/or its publication. This work was supported by the Zhejiang Provincial Disease Prevention and Control Science and Technology Program (Nos. 2025JK269 and 2026JKZ057), the Fourth Round of Ningbo Medical Key Discipline Construction Program (No. 2022-B18), and the Ningbo Top Medical and Health Research Program (No. 2023020713).

The original version of this article has been updated.

